# Upheaval in cancer care during the COVID-19 outbreak

**DOI:** 10.3332/ecancer.2020.ed97

**Published:** 2020-04-01

**Authors:** Omolola Salako, Kehinde Okunade, Matthew Allsop, Muhammedu Habeebu, Mariam Toye, Glory Oluyede, Gabriel Fagbenro, Babatunde Salako

**Affiliations:** 1 Department of Radiation Biology, Radiotherapy and Radiodiagnosis, College of Medicine, University of Lagos, Nigeria; 2 Oncological and Pathological studies Unit, Department of Obstetrics and Gynaecology, College of Medicine, University of Lagos, Nigeria; 3 Academic Unit of Palliative Care, University of Leeds, UK; 4 Health and Literacy Unit, Oumissa Inspire, Nigeria; 5 Department of Radiotherapy, NSIA-LUTH Cancer Centre, Nigeria; 6 X-Research Hub, Department of Radiotherapy, College of Medicine, University of Lagos, Nigeria

**Keywords:** COVID-19, cancer, coronavirus, cancer treatment

## Abstract

On Monday, 23 March 2020, Nigeria recorded its first mortality from the novel global COVID-19 outbreak. Before this, the country reported 36 confirmed cases (at the time of writing) and has discharged home two cases after weeks of care at a government-approved isolation centre in Lagos State. This first mortality was that of a 67-year-old man with a history of multiple myeloma, a type of blood cancer. He was undergoing chemotherapy and had just returned to Nigeria following medical treatment in the United Kingdom. The novel COVID-19 pandemic has grounded several global activities including the provision of health care services to people with chronic conditions such as cancer. Evidence from China suggests that cancer patients with COVID-19 infection are a vulnerable group, with a higher risk of severe illness resulting in intensive care unit admissions or death, particularly if they received chemotherapy or surgery. This letter is an attempt to suggest practicable interventions such as the use of existing digital health platforms to limit patients’ and oncology professionals’ physical interactions as a way of reducing the risk of COVID-19 infection transmission amongst cancer patients and oncologists, as well as outlining effective strategies to ensure that cancer care is not completely disrupted during the outbreak.

## Upheaval in cancer care during the COVID-19 outbreak in Nigeria

“The way we treat cancer over the next few months will change enormously…” writes a British Oncologist. “…It is highly probable that the cancer mortality and morbidity will rise, not because of the coronavirus pandemic, but because we are not able to treat cancers as we should normally [1].

In these uncertain times, we are having to make necessary adjustments to social distancing, large religious, academic and social activities are being shut down, with law enforcement agencies stationed in strategic places to ensure people do not gather as usual. A recent early morning drive on commercial routes and the ever congested Third Mainland Bridge and Ikorodu Road in Lagos state is smooth with low noise pollution and unusually free roads. The agile street entrepreneurs are present but look frustrated because they are unable to sell their wares and products, as there is neither traffic nor the consequent abundance of customers.

Novel Coronavirus, or COVID-19 infection, entered mainstream consciousness in December 2019 via the media when it first broke out in Wuhan, China. It is now becoming a stark global force for many, dominating political decision-making that has direct implications for our liberty. COVID-19 has disrupted economic activities around the globe such as trading and shipping and has ushered in a wave of scarcity and panic buying of essential products [2].

There are other problems including reduced sales revenue, pay cuts and job losses, and disruption of health care services. This is a global reality and Lagos state, the commercial hub of the country, has recorded 30 positive cases (at the time of writing) and discharged home two cases after they had received weeks of care whilst at a government-approved isolation centre in the State [3]. However, there is a strong possibility that there are undetected and asymptomatic cases; hence we may see a significant rise in the coming weeks. As expected, people with ill health will present to clinics and emergency wards, and health care workers will continue to be at the frontline of this pandemic whilst being at increased risk of exposure to the virus.

To control the transmission of coronavirus infection many clinical activities have been reduced. Suspicious COVID-19 cases are being identified, following a risk-assessment [4] while confirmed cases are referred or directly picked up for government- supervised isolation in approved facilities, by the Nigeria Centre for Disease Control (NCDC), autopsies are being deferred and some health care workers with a history of contact or exposure are now placed on home supervised self-quarantine.

Many ill patients too, with conditions including diabetes, cancer, and infectious diseases may be stranded at home or stuck on the wards of health facilities. Several clinical, social, academic and religious activities have ground to a halt. For those living with cancer, the diseases will continue to grow in the absence of treatment, therefore cancer may spread, develop resistance to treatments, cause multiple complications or, in some instances, death.

Reports from a nationwide study in China by Wenhua Liang stated that 18 participants out of 1,590 COVID-19 cases had a cancer history [5]. This group of patients, though small, recorded severe adverse events such as admission to the intensive care unit for invasive ventilation or death. These adverse events were most common in patients who had received chemotherapy or surgery within the last month. This study reported that a cancer history represented the highest risk for severe events especially in the elderly age group and clinical deterioration was rapid in this group.

The first COVID-19 death has been recorded in Nigeria. It was that of a 67-year-old man with a history of diabetes mellitus and multiple myeloma, a type of blood cancer. He was undergoing chemotherapy and he had just returned to Nigeria following medical treatment in the United Kingdom [6].

Our priority at this time should be to intensify public education amongst cancer patients and limit their risk of being infected as they fall into a vulnerable group.

## Effects of COVID-19 on cancer treatment

There is no doubt that the delivery of cancer care will be disrupted. Cancer clinics may need to reduce clinical appointments, chemotherapy administration encounters, alter radiotherapy schedules, and cancel or postpone elective cancer surgeries. There may be a shortage of supplies, non-availability of drugs and consumables. Newly diagnosed and existing patients with cancer who experience chest symptoms (e.g. breathlessness) due to lung metastases may be denied care due to heightened suspicion of COVID-19 infection. For patients with cancer, these will have huge repercussions on their experience and management of cancer-related symptoms, quality of life and survival.

According to the American Society of Clinical Oncology(ASCO), “There is no direct evidence to support changes in cancer regimen during the pandemic” [7]. Therefore, routinely stopping anticancer or immunosuppressive therapy is not recommended. There are currently cancellations or postponement of elective cancer surgeries and in the coming days radiotherapy appointments may also be affected. Community-based palliative treatment of advanced cancers will also be affected significantly as health care workers are minimising their contacts through social distancing and due to the increase in demand for their expertise in the treatment of confirmed cases in the health facilities. Thus, there is a grave impact on disease control.

For us oncology professionals working in low-resource settings, we are faced with the dilemma of striking a balance between protecting ourselves and giving our patients a good chance to fight their cancers during the COVID-19 outbreak. We must tread with caution, as we are a critical workforce, highly trained and skilled in treating cancer, as well as being an endangered specialty with a limited human resource pool in the country. This is not the time to be careless in the frontline as we are all at risk and we must do all that is required to reduce our chances of getting infected. The World Health Organization’s recommendation for health care providers in this COVID-19 era is well articulated in an online resource [8].

## How do we respond?

1. **Develop a COVID 19 policy for cancer care: **Establish an oncology multidisciplinary committee to create, review and rapidly update a clinical policy suitable to our setting.
  Provide cancer patients with a cover note that enables seamless movement if they get stopped by law enforcement agents on the roads.
2. **Strategise to reduce clinic visits and accelerate remote care: **Because cancer patients and caregivers are expectedly anxious whether they are symptomatic or not, and their genuine desire for improved survival; they will require information, counselling, symptomatic control and treatment. Traditionally this is achieved through hospital visits but can be achieved by telemedicine. It is a matter of urgency that we accelerate remote cancer care. Oncology centres should within 72 hours transit to delivering 30-50% of services online.
3. **Empower patients and caregivers through communication: **Leverage on digital technologies to ensure we continue to communicate, to patients and with each other. Bulk text messaging, email and or voice calls can ensure we provide patients with clear communication about how the hospital is responding to and preventing COVID-19 infection and how the infection will affect cancer care going forward. This will keep them abreast of happenings and allay their fears. For example, establish a clinical call centre manned by administrative and clinical staff knowledgeable in the provision of cancer care and support services. This category of responders must be trained in client service and people relations as the bulk of the calls would be from agitated and worried patients. This is so that the staff do not react in untoward ways to the emotion-laden calls they are bound to receive.
4. **Leverage existing digital health platforms: **Cancer centres should integrate existing digital health platforms to limit patients and oncology professional’s physical interaction, a strategy that several oncology centres in the USA are adopting [9]. Digital platforms such as www.oncopadi.com already exist and were designed for low-resource settings. They can provide telemedicine services, consultations, symptom control and counselling to cancer patients and their caregivers. To facilitate the use of telehealth solutions across all contexts, a global directory of telehealth platforms is also being compiled by digital health experts which outlines multiple platforms (many are free) to support care delivery during the COVID-19 outbreak: https://docs.google.com/spreadsheets/d/1XMsJJIduO6yI_GEo1Vy_b_SXoz9YwbgtEL63-siNS_Q/edit#gid=0.
5. **Administer: **Continue providing only emergencies and essential services such as radiotherapy under established safety protocols.
6. **Switch: **from systemic therapy to oral chemotherapy, and monitor side effects remotely. This should be discussed and agreed upon by a multidisciplinary team and communicated with patients only if the pandemic stops patients from visiting the hospital. This may be a better option than no treatment at all.
7. **Practice: **the World Health Organization’s advice on reducing coronavirus transmission for the public [10].

### Palliative cancer care during the COVID-19 outbreak

Palliation is an ethical obligation and we should strive for good symptom control and comfort care for cancer patients in a pandemic [11]. To support palliative and supportive care of people with cancer, these proposed four areas (4S) for palliating a pandemic [11] can be used to guide a response but need to be adapted and considered in the context of the availability of resources and expertise in the context of low-resource settings:

- “Stuff” (i.e. stockpile of medications, equipment to deliver medications)
- “Staff” (identifying and consulting with clinicians with palliative care expertise, deliver focused education sessions to frontline staff for symptom management and supporting end-of-life care for patients with COVID-19)
- “Space” (e.g. identify wards and nonclinical areas in all health care facilities that would be appropriate to accommodate large numbers of patients expected to die)
- “Systems” (e.g. develop systems for direct consultation of palliative care expertise for staff in hospitals, create a triage system to identify patients with cancer in need of specialist palliative care management)

Bereavement needs to be considered in the context of COVID-19 too. Globally we are seeing increasing numbers of unexpected and premature deaths from COVID-19. Families will need support with bereavement where they may have been expecting a longer trajectory of illness for a loved one with cancer. Furthermore, traditional cultural rituals and ceremonies may be prohibited in line with social distancing. We need talk to spiritual leaders to understand what can occur and provide guidance to the families of those who die to discuss how and when they might be able to hold services for their loved ones.

A time like this is a true test of leadership and our values. As leaders we must make clinical and economical decisions in line with the ethical rationing of resources and the global call to flatten the curve of the infection.

## Conclusion

To control the transmission of coronavirus infection many clinical activities have been reduced in most countries, including Nigeria. For people living with cancer, the diseases will continue to grow in the absence of treatment, therefore cancer may spread, develop resistance to treatments, cause multiple complications or, in some instances, death. To respond to this important area of critical need, government must constitute a multidisciplinary oncology and infectious disease team to create, review and rapidly update a clinical policy for combating COVID-19 infection during cancer care, strategise to reduce clinic visits and accelerate remote online care, empower patients and caregivers through communication using digital technology interventions and leverage on existing digital health platforms in the country. In addition, efforts should be made to support palliative and supportive care of people with advanced cancer during the outbreak by systematically identifying critical areas for consideration and development in the context of the availability of resources and expertise in resource-constrained settings such as Nigeria.

## Funding statement

The authors declare that they have no funding for this article.

## Conflicts of interest

The authors declare that they have no conflicts of interest regarding this work.
